# Integrated Multilayer Omics Reveals the Underlying Mechanisms in Xylazine-Related Heart Injury in Rats

**DOI:** 10.3390/ijms26178532

**Published:** 2025-09-02

**Authors:** Yangchang Ou, Tingting Mai, Ning Wang, Zhiyan Li, Yangchang Chen, Shuquan Zhao

**Affiliations:** Faculty of Forensic Medicine, Zhongshan School of Medicine, Sun Yat-sen University, Guangzhou 510080, China; ouych8@mail2.sysu.edu.cn (Y.O.); maitt@mail2.sysu.edu.cn (T.M.); wangn265@mail2.sysu.edu.cn (N.W.); lizhiy27@mail.sysu.edu.cn (Z.L.); chenych53@mail.sysu.edu.cn (Y.C.)

**Keywords:** xylazine poisoning, multi-omics analysis, fructose and mannose metabolism, cholesterol metabolism

## Abstract

Xylazine abuse is emerging as a global problem, whereas the toxic mechanisms of xylazine poisoning are seldom studied. The present study aims to assess the heart injury in xylazine poisoning and uncover the underlying mechanism. Forty male SD rats were randomly dived into four groups: control (saline), low dose (10 mg/kg xylazine), median dose (20 mg/kg xylazine) and high dose (40 mg/kg xylazine). The rats were injected with the drug intraperitoneally for 28 consecutive days, and then cardiac ultrasound examination was performed and serum and heart tissues were collected. Genomic, proteomic, and metabolic omics analyses were conducted. ELISA, RNA sequencing, histopathology examination, RT-qPCR, and Western blot were performed. Repeated injection of xylazine led to a decrease in the expression of cardiac output (CO), ventricular systole (VS), and ventricular diastole (VD), while concurrently elevating the levels of lactate dehydrogenase (LDH), creatine kinase myocardial band (CK-MB), and cardiac troponin T (c-TNT) in the serum. HE staining analysis showed evidence of contraction band necrosis, interstitial fibrosis, and infiltration by inflammatory cells in animals with xylazine poisoning. The modified genes, proteins, and metabolites were gathered, and the integration of transcriptomic, proteomic, and metabolic networks identified 25 overlapping pathways between the differentially expressed genes and metabolites (DEGs-DEMs) and the differentially expressed proteins and metabolites (DEPs-DEMs) joint pathways. The majority of these pathways pertained to the metabolism of sugars, amino acids, and fats. The proteins associated with fructose and mannose metabolism, as well as cholesterol metabolism, were validated, thereby substantiating their pivotal role in the development of xylazine-induced cardiac injury. Repeated injection of xylazine impaired heart function and the metabolism of fructose and mannose. Cholesterol metabolism pathways were critical in the process of xylazine-induced heart injury.

## 1. Introduction

Xylazine, an agonist targeting α2 adrenergic receptors, is frequently employed in veterinary settings. Nevertheless, since 2019, it has gained traction among addicts because it allows for a lower dosage of fentanyl or opioids while extending the duration of the effects when used in combination with these drugs [1,2,3]. Presently, its usage is widespread in the United States and is increasingly internationally [4]. In 2024 and 2025, a case involving a xylazine-linked fatality was documented through forensic autopsy in Europe and China [5,6]. Owing to its pharmacological attributes, xylazine can impair the central nervous system, leading to conditions such as coma, bradycardia, and hypotension [7]. Previous studies confirmed bradycardia and hypotension were the common findings in xylazine poisoning, whereas the exact mechanisms behind this phenomenon are still not fully illustrated [8].

With technological progress, the fields of genomics, proteomics, and metabolomics have been incorporated into medical practice to elucidate the mechanisms underlying various diseases and to pinpoint potential biomarkers [9,10,11]. It has been confirmed that these tools are useful in exploring the underlying mechanisms of various toxic substances. In 2022, Yan et al. utilized multi-omics, including genomics, proteomics, and metabolomics, to explore the mechanisms underlying the role of histidyl dipeptides in the heart and confirmed the critical role of histidyl dipeptides in the structure, function, and energetics of the heart [10]. In 2022, Léa et al. employed multi-omics profiling, which included methylome, transcriptome, proteomic, and metabolite data, to clarify the characteristics of environmental exposures during childhood and to reveal the signatures of toxic chemical compounds, thereby providing a valuable reference for further research [10].

Although heart toxicity in has been reported in people with xylazine poisoning, the underlying mechanisms are far from being fully understood. Most studies have focused on a single overdose of xylazine, with cardiac changes following a long period of xylazine injections being largely unexplored [6,7,8]. Given the emergence of xylazine as a public health issue, it is urgent to investigate the effects of prolonged xylazine injections on the heart and clarify the underlying mechanisms. Therefore, we used a 28-day xylazine injection rat model based on our previous study and conducted multi-layer omics analyses to explore genomic, proteomic, and metabolic changes, thereby providing a reference for clinical practice.

## 2. Results

### 2.1. Long-Term Injection of Xylazine in Rats Induced Heart Injury

**Figure 1:**
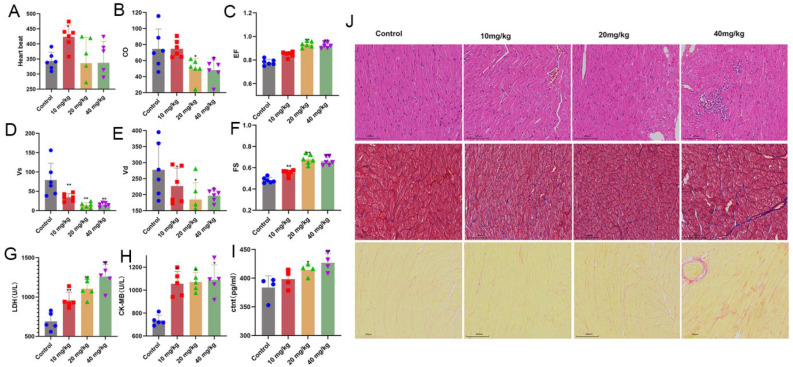
Repeated injection of xylazine decreases the function of the heart and causes heart injury. (**A**) The heartbeat changes associated with repeated xylazine injection. (**B**) Repeated xylazine injection decreased the CO of the rats. (**C**) Repeated xylazine injection increased the EF of the rats. (**D**) Repeated xylazine injection decreased the Vs of the rats. (**E**) Repeated xylazine injection decreased the Vd of the rats. (**F**) Repeated xylazine injection increased the FS of the rats. (**G**). Repeated xylazine injection increased the level of LDH in the serum. (**H**). Repeated xylazine injection increased the level of CK-MB in the serum. (**I**) Repeated xylazine injection increased the level of c-Tnt in the serum. (**J**) The pathological changes that occurred due to repeated xylazine injection, HE, 100×, Masson, 100×, Sirius Red, 200×. *, *p*  <  0.05 compared with the control group. **, *p*  <  0.01 compared with the control group.

As shown in Figure 1, repeated injections of xylazine increased the ejection fraction (EF) and fractional shortening (FS), while decreasing cardiac output (CO), ventricular systole (VS), and ventricular diastole (VD). Additionally, the serum levels of lactate dehydrogenase (LDH), creatine kinase myocardial band (CK-MB), and cardiac troponin T (c-TNT) were elevated with increasing doses of xylazine. Pathological examination displayed contraction band necrosis, interstitial fibrosis, and inflammatory cell infiltration confirmed the presence of xylazine-induced heart injury, and it became more pronounced with the increase in doses of xylazine.

### 2.2. Identification of DEGs in Repeated Xylazine Injection-Induced Heart Injury

All samples were sequenced using the BGISEQ platform, producing 6.67 Gb of data per sample. Each sample yielded 45.44 million total raw reads and over 43.98 million total clean reads. The Q20 percentage of the clean reads was at least 97.95%, and the Q30 percentage was at least 97.02%. The average mapping rate to the reference genome was 95.58%, while the average mapping rate to genes was 56.51%. A total of 18,451 genes were identified. Subsequently, the data underwent further analysis.

**Figure 2:**
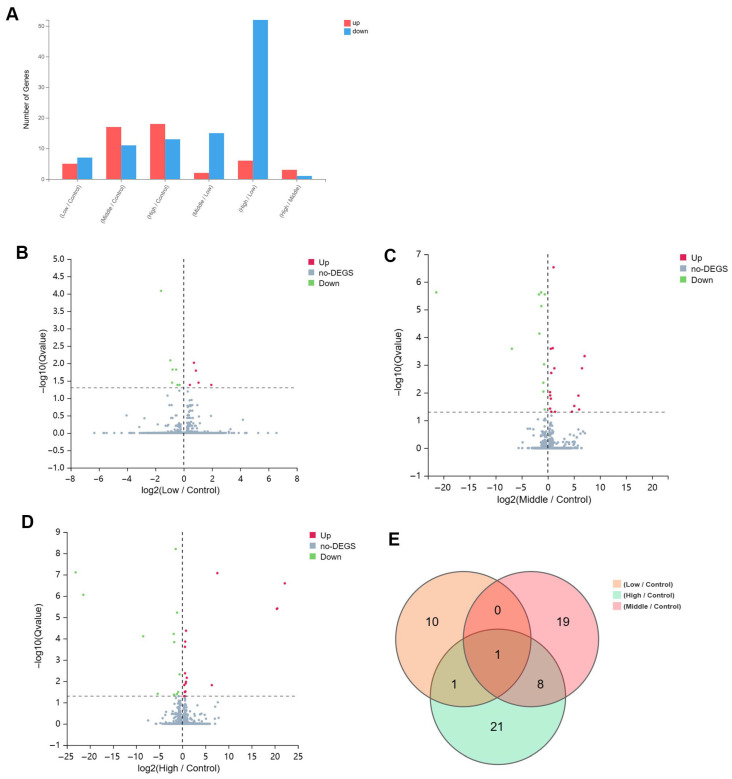
The basic information of the DEGs in repeated xylazine injection-induced heart injury, and the number of altered genes increased with the exposure dose of xylazine. (**A**) The numbers of DEGs in various groups. (**B**) The volcano plot of 10 mg/kg vs. the control group. (**C**) The volcano plot of 20 mg/kg vs. the control group. (**D**). The volcano plot of 40 mg/kg vs. the control group. (**E**) Venn diagrams of the DEGs in instances of repeated xylazine injection-induced heart injury.

As the dosage of xylazine increased, the number of differentially expressed genes (DEGs) also rose. In the 40 mg/kg group, 18 genes were up-regulated and 13 down-regulated compared to the control group. The 20 mg/kg group exhibited 17 up-regulated and 11 down-regulated genes relative to the control. In the 10 mg/kg group, there were five up-regulated and seven down-regulated genes compared to the control. A common gene, Dbp, was down-regulated in all injection groups compared to the control. Additionally, eight common DEGs were identified in both the 20 mg/kg and 40 mg/kg groups compared to the control (Figure 2).

**Figure 3:**
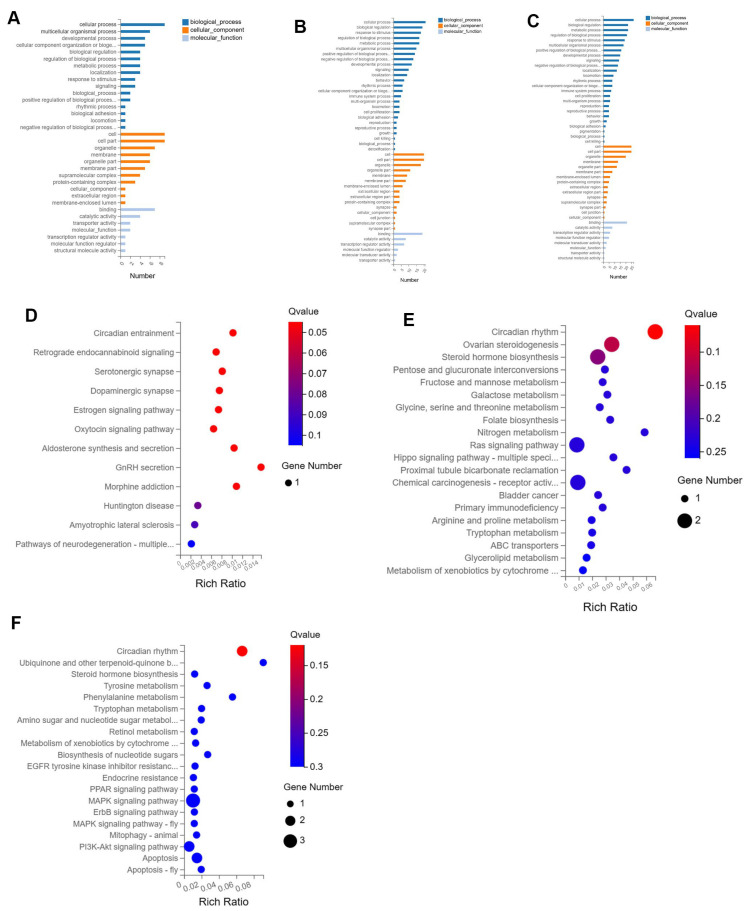
GO and KEGG enrichment analysis of the DEGs in repeated xylazine injection-induced heart injury. (**A**) GO enrichment analysis of the DEGs of 10 mg/kg vs. the control group. (**B**) GO enrichment analysis of the DEGs of 20 mg/kg vs. the control group. (**C**) GO enrichment analysis of the DEGs of 40 mg/kg vs. the control group. (**D**) KEGG enrichment analysis of the DEGs of 10 mg/kg vs. the control group. (**E**) KEGG enrichment analysis of the DEGs of 20 mg/kg vs. the control group. (**F**) KEGG enrichment analysis of the DEGs of 40 mg/kg vs. the control group.

The Gene Ontology (GO) enrichment analysis revealed that as the injection dose of xylazine increased, the involvement of biological processes also escalated, with cellular processes being the most prominent across all groups (Figure 3). Biological regulation was the second most affected in both the 20 mg/kg and 40 mg/kg groups compared to the control. Subsequently, KEGG pathway analysis was conducted, and the top 20 affected pathways were compiled to create the bubble charts. The circadian rhythm pathway was the most affected in all groups. The RNA sequencing results suggested that the repeated administration of xylazine altered the genetic profile of the heart.

### 2.3. Proteomic Changes in Repeated Xylazine Injection-Induced Heart Injury

**Figure 4:**
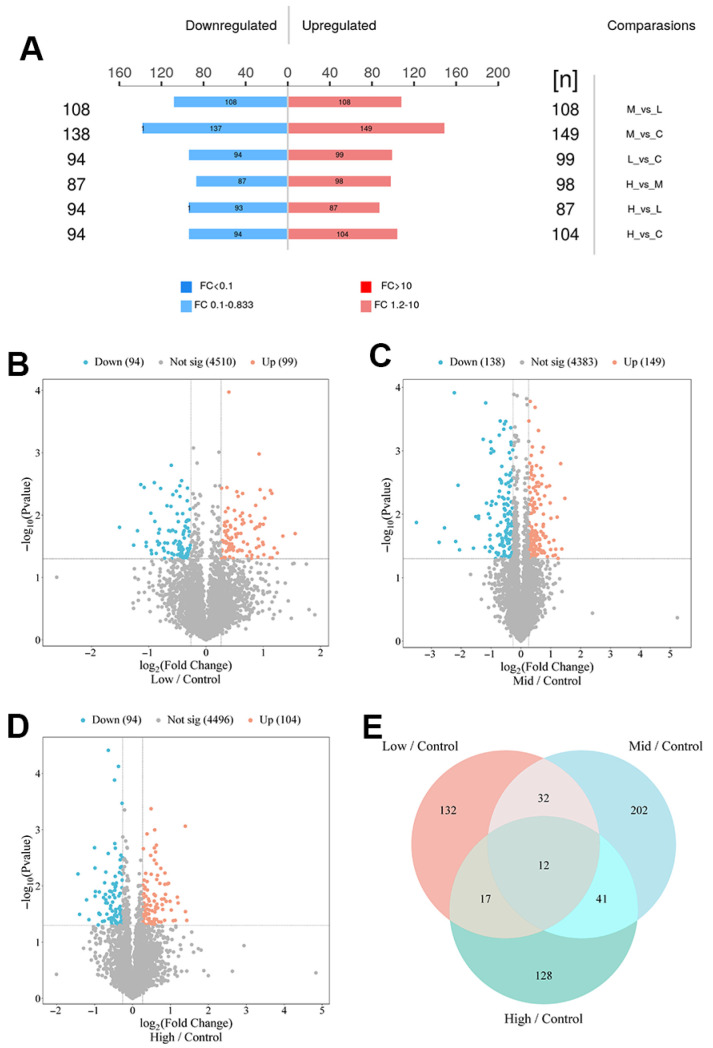
Basic information about the DEPs in repeated xylazine injection-induced heart injury, and the number of DEPs increased with the exposure dose of xylazine. (**A**) Numbers of DEPs in various groups. (**B**) Volcano plot of 10 mg/kg vs. the control group. (**C**) Volcano plot of 20 mg/kg vs. the control group. (**D**) Volcano plot of 40 mg/kg vs. the control group. (**E**) Venn diagrams of the DEPs in repeated xylazine injection-induced heart injury.

Although the flow of information from RNA to protein translation is considered the central dogma, numerous studies have shown a weak correlation between mRNA expression and the abundance of translated proteins. To determine whether differentially expressed genes (DEGs) in repeated xylazine injection groups are translated to the protein level, we examined the protein profiles of cardiac tissues from repeated xylazine injection groups and analyzed the differences in protein expression levels through proteomic analysis. In all groups, over 4874 different proteins were identified. Differentially expressed proteins (DEPs) were identified based on fold changes of *p* < 0.05 and ≥1.2 between all groups. It was revealed that there were 94 down-regulated and 99 up-regulated proteins in the 10 mg/kg group compared to the control group. In the 20 mg/kg group, there were 138 down-regulated and 149 up-regulated proteins, and in the 40 mg/kg group, there were 94 down-regulated and 104 up-regulated proteins. Additionally, 12 common proteins were observed in all repeated xylazine injection groups (Figure 4).

**Figure 5:**
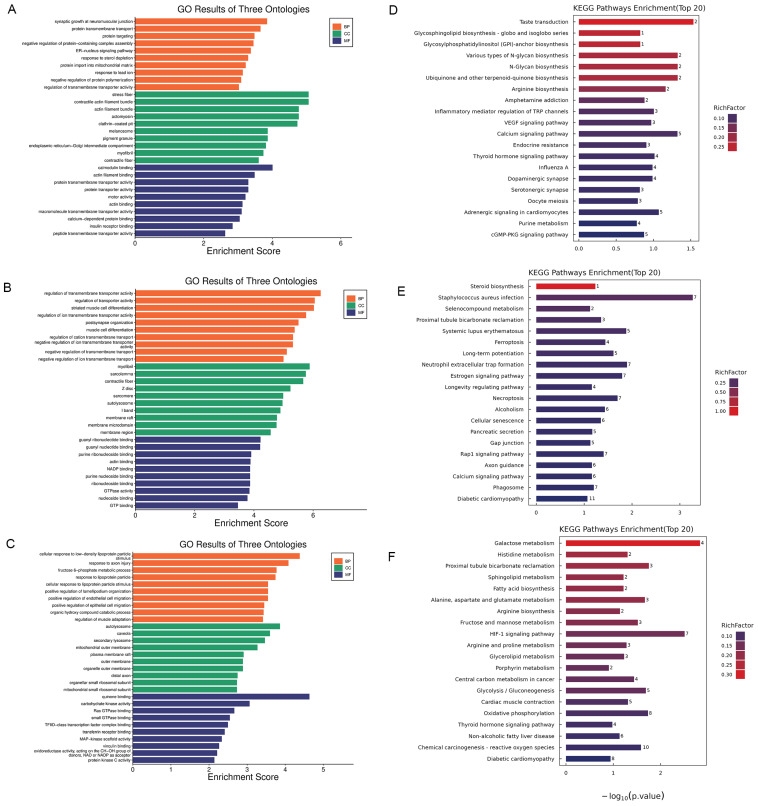
The GO and KEGG enrichment analysis of the DEPs in repeated xylazine injection-induced heart injury. (**A**) GO enrichment analysis of the DEPs of 10 mg/kg vs. the control group. (**B**) GO enrichment analysis of the DEPs of 20 mg/kg vs. the control group. (**C**) GO enrichment analysis of the DEPs of 40 mg/kg vs. the control group. (**D**) KEGG enrichment analysis of the DEPs of 10 mg/kg vs. the control group. (**E**) KEGG enrichment analysis of the DEPs of 20 mg/kg vs. the control group. (**F**) KEGG enrichment analysis of the DEPs of 40 mg/kg vs. the control group.

To identify changes in specific biochemical pathways, we annotated the differentially expressed proteins to the NCBI annotation system, Gene Ontology (GO). The GO enrichment analysis revealed that biological processes were crucial in the process of xylazine-induced heart injury (Figure 5). Within the biological processes, cellular processes, biological regulation, regulation of biological processes, and metabolic processes were the most affected. Regarding Cellular Component, cellular anatomical entities and protein-containing complexes were the most impacted groups. Binding and catalytic activities were the top-ranked categories in Molecular Function. Subsequently, we performed KEGG pathway analysis and generated bubble charts depicting the top 20 affected pathways. As illustrated in the figure, various metabolism pathways were altered in rats receiving different doses of xylazine, indicating that repeated xylazine injections modified the metabolic processes of the rats.

### 2.4. Metabolic Changes in Repeated Xylazine Injection-Induced Heart Injury

To better understand the metabolic changes associated with repeated xylazine-induced heart injury, we compared the serum metabolic profiles of rats administered varying doses of xylazine. Raw data was collected using LC-MS/MS for peak extraction and identification, yielding the peak areas and identification results of metabolites. The base peak chromatograms of all samples indicated the instrument’s good condition and the stability of the signal throughout the process. After selecting all quality control (QC) samples from the entire set, a Pearson’s correlation analysis was conducted. A higher correlation among QC samples (with R values closer to 1) signifies the greater stability of the testing process and higher data quality.

**Figure 6:**
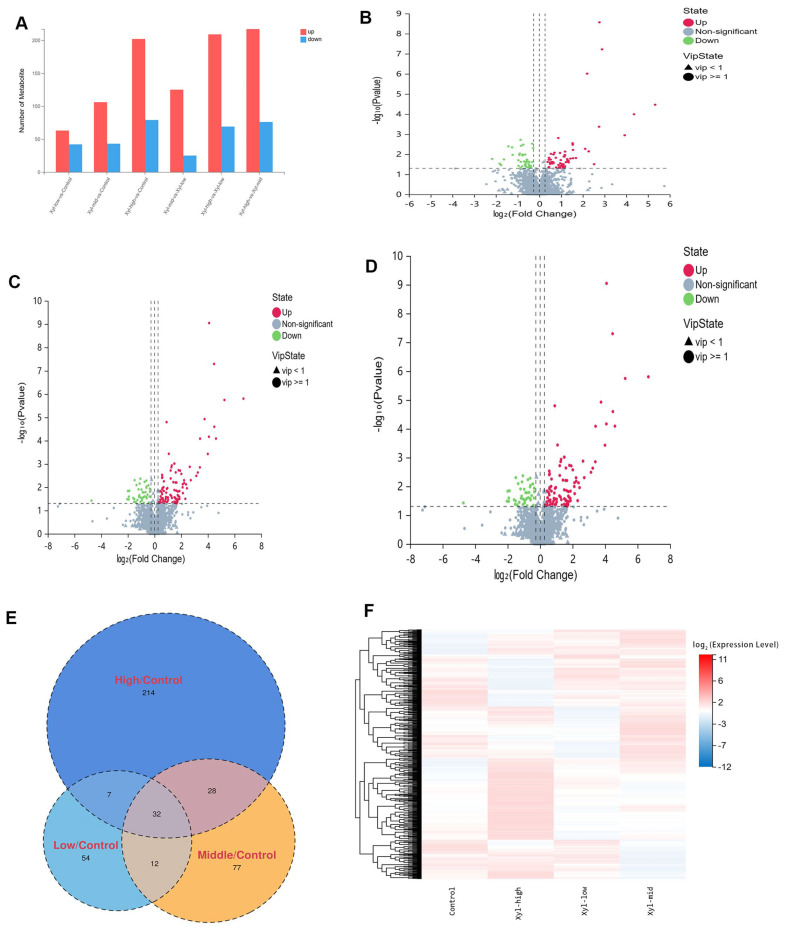
The basic information of the DEMs in repeated xylazine injection-induced heart injury; the number of DEMs increased with the exposure dose of xylazine. (**A**) Numbers of DEMs in various groups. (**B**) Volcano plot of 10 mg/kg vs. the control group. (**C**) The volcano plot of 20 mg/kg vs. the control group. (**D**) The volcano plot of 40 mg/kg vs. the control group. (**E**) Venn diagrams of the DEMs in repeated xylazine injection-induced heart injury. (**F**) Mean heatmap of the DEMs in repeated xylazine injection-induced heart injury.

A total of 4189 metabolites were detected, and 1694 were identified. Amino acid metabolism constitutes 28.3% of all metabolisms. It was observed that as the injection doses of xylazine increased, the number of metabolites that changed also increased (Figure 6). In the 10 mg/kg group, there were 63 up-regulated and 42 down-regulated metabolites compared to the control group. In the 20 mg/kg group, there were 106 up-regulated and 43 down-regulated metabolites. In the 40 mg/kg group, there were 202 up-regulated and 79 down-regulated metabolites. Additionally, 32 common metabolites were observed across all xylazine repeated injection groups. The PCA and PLS-DA results indicated that changes in injection doses altered the metabolites and could be further analyzed.

**Figure 7:**
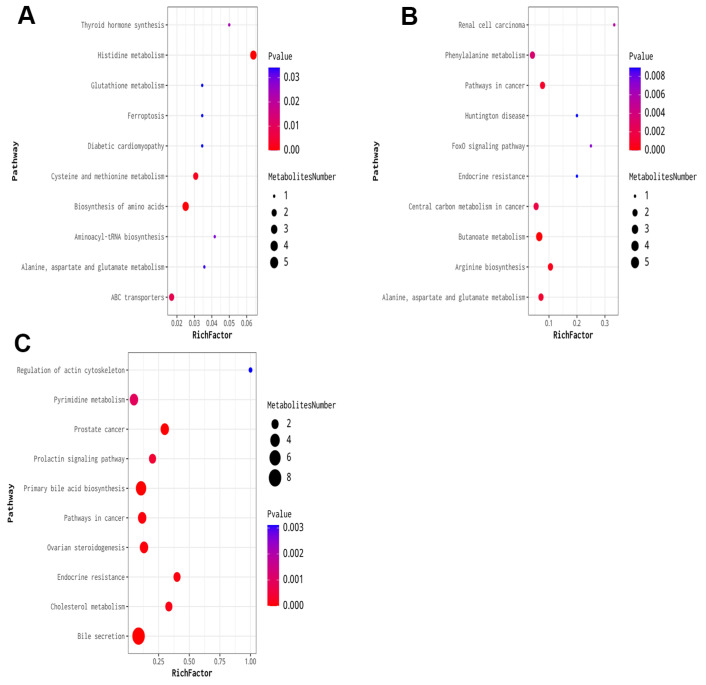
KEGG enrichment analysis of the DEMs in repeated xylazine injection-induced heart injury. (**A**) KEGG enrichment analysis of the DEMs of 10 mg/kg vs. the control group. (**B**) KEGG enrichment analysis of the DEMs of 20 mg/kg vs. the control group. (**C**) KEGG enrichment analysis of the DEMs of 40 mg/kg vs. the control group.

Cluster analysis of the expression levels of differential metabolites across multiple comparison groups provides a direct visualization of the metabolite expression in each group’s samples. This analysis revealed that the metabolites were altered in various groups. KEGG pathway analysis indicated that the altered metabolites were enriched in specific pathways (Figure 7). In the 10 mg/kg groups compared to the control, the top ten affected pathways included histidine metabolism, biosynthesis of amino acids, cysteine and methionine metabolism; ABC transporters; thyroid hormone synthesis; aminoacyl-tRNA biosynthesis; alanine, aspartate, and glutamate metabolism; diabetic cardiomyopathy; ferroptosis; and glutathione metabolism. For the 10 mg/kg groups compared to the control, the top three affected pathways were histidine metabolism, biosynthesis of amino acids, and cysteine and methionine metabolism. In the 20 mg/kg groups, the top three affected pathways were butanoate metabolism, arginine biosynthesis, and pathways in cancer. Finally, in the 40 mg/kg groups compared with the control, the top three affected pathways were bile secretion, primary bile acid biosynthesis, and prostate cancer.

### 2.5. Integration of the Transcriptomic, Proteomic, and Metabolic Networks

**Figure 8:**
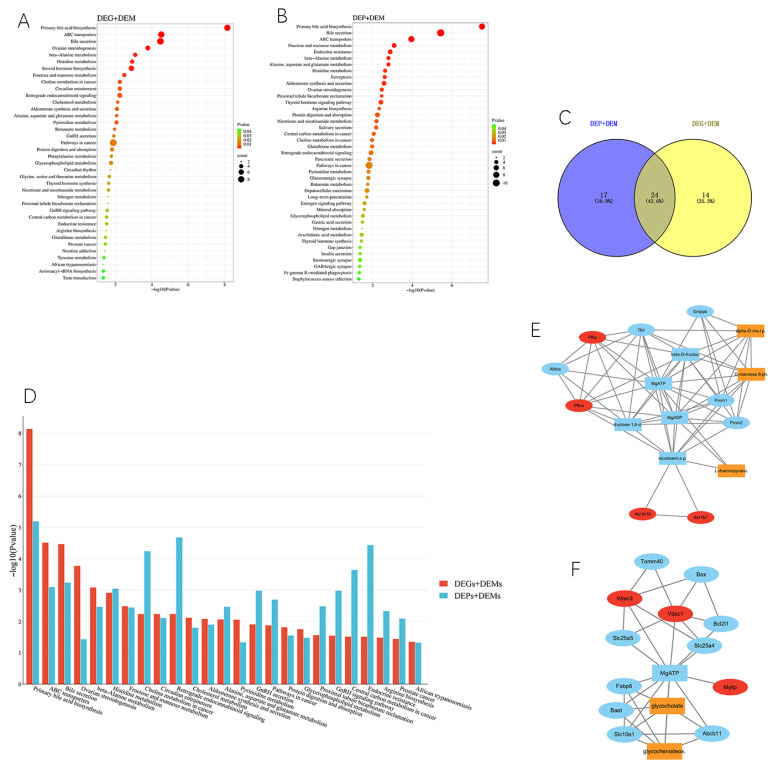
Integrative analysis of transcriptomics, proteomics and metabolomics. (**A**) KEGG pathway analysis between DEGs and DEMs. (**B**) KEGG pathway analysis between DEPs and DEMs. (**C**) Venn plot of DEGs-DEMs and DEPs-DEMs. (**D**) The overlapped KEGG pathway. (**E**) PPI of the fructose and mannose metabolism signaling pathway. (**F**) The PPI of the cholesterol metabolism signaling pathway.

The integrative analysis indicated a total of 38 differentially expressed genes (DEGs) and differentially expressed metabolite (DEM) joint pathways and 109 differentially expressed protein (DEP) and DEM joint pathways under a condition of *p* < 0.05 between the xylazine and control groups. The common pathways in DEGs-DEMs and DEPs-DEMs were further analyzed, revealing 25 overlapped pathways between the two joint pathways. This suggested that xylazine could affect these metabolic pathways by simultaneously regulating genes, proteins, and metabolites. The overlapped pathways were shown in Figure 8 and most of them were related to sugars, amino acids, and fat metabolism. The fructose and mannose metabolism, as well as the cholesterol metabolism pathways, were considered to play a critical role in the process of xylazine-induced heart injury. A protein–protein interaction (PPI) database was used to select the hub targets in these pathways, and the interaction network displayed the affected hub targets in the pathways. In the cholesterol metabolism pathways, xylazine affected the production of glycocholate and glycochenodeoxycholate through vdac3, vdac1, and mylip. Xylazine also affected the production of alpha-D-mannose 1-phosphate, D-mannose 6-phosphate, and L-rhamnopyranose through pfkm, pfkp, Akr1b7, and Akr1b10 (Figures S1 and S2).

### 2.6. Validation of the Pathway

**Figure 9:**
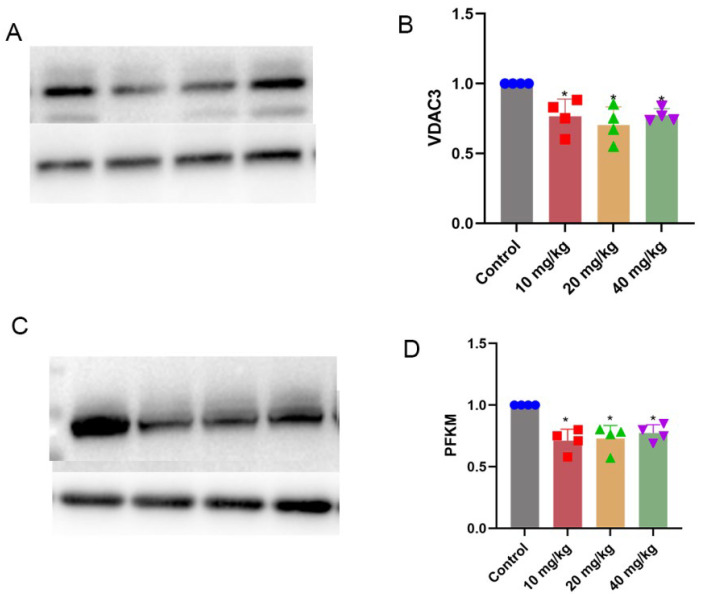
Validation of related proteins in the fructose and mannose metabolism signaling pathway and cholesterol metabolism signaling pathway. (**A**) Western blot image of VDAC3. (**B**) Quantification of VDAC3. (**C**) Western blot image of PFKM. (**D**) Quantification of PFKM. * *p*  <  0.05 compared with the control group.

The expression levels of vdac3 and pfkm were decreased in the xylazine injection groups (Figure 9).

## 3. Discussion

The present study confirmed that repeated exposure to xylazine induced heart injury through cardiac ultrasound, cardiac enzymes, and histopathological examination. The altered genes, proteins, and metabolites were meticulously described through transcriptomic, proteomic, and metabolic analyses. The integration of these multi-omics networks revealed overlapping pathways between differentially expressed genes and metabolites (DEGs-DEMs) and differentially expressed proteins and metabolites (DEPs-DEMs). Most of these pathways were related to the metabolism of sugars, amino acids, and fats, confirming the crucial role of energy metabolism in xylazine-induced heart injury. Proteins involved in fructose and mannose metabolism, as well as cholesterol metabolism pathways, were verified, thereby confirming their critical role in the process of xylazine-induced heart injury.

Coma, bradycardia, and hypotension are the primary symptoms of xylazine poisoning, which may manifest within 30 min and can persist for several days [12]. To date, no specific pathological changes have been identified in fatal cases or autopsy samples from people with xylazine poisoning [13,14]. Previous animal experiments confirmed that xylazine not only slows the heart rate, but also increases the inotropic effect in animals, and it can be blocked by the α receptor antagonist yohimbine in vitro, whereas yohimbine failed to reverse the effect of xylazine in ex vivo heart experiments, indicating that xylazine has a direct effect on the heart itself [15]. With the increase in xylazine exposure, the decreased CO, VS, and VD, and increased serum levels of LDH, CK-MB, and c-TNT confirmed that xylazine exposure hurt the cardiac function. Combined with pathological examination of phenomena such as contraction band necrosis, interstitial fibrosis, and inflammatory cell infiltration, it was confirmed that repeated xylazine exposure causes heart injury.

To clarify the underlying mechanisms of xylazine injection-induced heart injury, multi-omics were conducted. The omics results revealed that the number of different expression genes, proteins and metabolites increased with the xylazine injection dose. In RNA sequencing results, the role of biological processes increased and cellular processes ranked first in all groups in the GO enrichment analysis. Circadian rhythm ranked top in all groups in the KEGG pathway analysis. Previous studies that indicated circadian rhythm plays a crucial role in the process of heart failure and targeting it is a potential therapeutic strategy [16,17,18]. With the increase in the injection dose, the role of energy metabolism became more important in the process of xylazine injection-induced heart injury. Generally, the RNA sequencing results indicated that the repeated injection of xylazine changed the genetic profile of the heart.

It is widely recognized that transcriptomics reveals mechanisms by examining gene expression; however, this approach alone is insufficient to elucidate biological functions. Consequently, we conducted proteomic analyses to further investigate the underlying mechanisms through the expression of proteins. Typically, repeated administration of xylazine alters the protein profile of the heart. In all groups receiving repeated xylazine injections, 12 common proteins were observed. GO enrichment analysis revealed that biological processes played a crucial role in the development of xylazine-induced heart injury. Subsequently, we performed KEGG pathway analysis, which indicated that various metabolic pathways were affected in the different dose groups of xylazine-injected rats, suggesting that repeated xylazine injections modify the metabolism of these animals.

The metabolomics analysis was conducted to verify the results of the transcriptomics and proteomics studies. A total of 1694 metabolites were identified, with amino acid metabolism accounting for 28.3% of all metabolites. The analysis revealed that with the increased doses of xylazine exposure, DEMs were also increased. Thirty-two common metabolites were observed in all groups. The KEGG pathway analysis indicated that the main pathways affected in the repeated injection groups were histidine metabolism, biosynthesis of amino acids, cysteine and methionine metabolism, butanoate metabolism, arginine biosynthesis, phenylalanine metabolism, cholesterol metabolism, and bile secretion.

To better understand the mechanisms of xylazine-induced heart injury, we integrated transcriptomic, proteomic, and metabolic results and conducted multi-omics analysis. We confirmed 25 overlapping pathways between DEGs and DEMs, as well as between DEPs and DEMs. Most of these pathways were related to the metabolism of sugars, amino acids, and fats. The fructose and mannose metabolism and cholesterol metabolism pathways were considered to play a critical role in the process of xylazine-induced heart injury. It is commonly understood that long-chain fatty acids are the energy supply in normal conditions, whereas carbohydrate metabolism becomes the primary source in an enlarged heart [19,20,21]. Cardiac hypertrophy increases energy expenditure, decreases redox capacity, and damages coronary arteries, resulting in myocardial ischemia [20]. Ischemia shifts energy metabolism from aerobic to anaerobic metabolism. Glycolysis becomes the primary source of ATP in ischemic myocardium, and high levels of NADH and FADH2 inhibit β-oxidation, leading to the accumulation of long-chain acyl-CoA and acylcarnitine esters [22,23,24]. Previous studies have confirmed the crucial role of lipids in the energy production of the heart; alterations and imbalances in lipid oxidation affect the heart’s energy production and thus play a role in the process of heart failure [19,20,21].

Mitochondrial homeostasis is crucial in energy production and the essential voltage-dependent anion channel 1 (VDAC1 and VDAC3) membrane proteins of the mitochondria are critical for mitochondrial function. A previous study also confirmed that a decrease in VDAC3 in sepsis-induced myocardial injury and overexpression of VDAC3 reduce mitochondrial oxidative stress, reverse the progression of ferroptosis, and thus play a protective role in sepsis-induced myocardial injury [25]. A recent study indicated that PFKM is a key enzyme for glycolytic activation and it accelerates cardiac fibroblasts and thus causes atrial fibrillation [26]. The changed VDAC3 and PEKM protein indicated cardiac metabolic reprogramming in a repeated-xylazine-exposure heart, and suggested that the fructose and mannose metabolism and cholesterol metabolism pathways play a crucial role in the process of xylazine-induced heart injury.

## 4. Materials and Methods

### 4.1. Animals and Treatment

The repeated administration of the xylazine animal model was based on published work by Yi et al. [27]. Forty male Sprague-Dawley (SD) rats were randomly assigned to four groups: a control group (receiving normal saline), a low-dose group (administered 10 mg/kg of xylazine), a medium-dose group (20 mg/kg of xylazine), and a high-dose group (40 mg/kg of xylazine). They were injected with the respective substances continuously for 28 days. And a day later, cardiac ultrasound was performed to evaluate the cardiac changes in these animals and then they were sacrificed to collect the heart tissue from the apex of the left ventricular, along with blood samples, for further study. All experimental procedures were approved by the Animal Care and Use Committee of Sun Yat-sen University (SYSU-IACUC-2024-B0804) and adhered to the ethical guidelines for experimental animals in China.

### 4.2. Cardiac Ultrasound Examinartion

The rats were anesthetized with isoflurane and fixed on the platform, and the electrodes were coated with conductive paste and attached to the rat’s paws to collect the correct ECG. The small animal ultrasound imaging system Vevo3100 was used to perform the cardiac ultrasound examination of the rats. The fur from the chest and abdomen was removed and M-Mode images were collected for further analysis.

### 4.3. Biochemical Analysis

LDH, CK-MB and cTNT in the Serum

The whole blood were placed at room temperature for 2 h and then centrifuged at 4 °C 3000/min for 15 mins and then stored at −80 °C for further examination. LDH and CK-MB were quantified using assay kits (Elabscience, Wuhan, Hubei, China).

### 4.4. Histopathological Examination

HE staining was conducted to evaluate the heart pathology changes. The rat hearts were fixed with 4% paraformaldehyde for more than 24 h and dehydrated with a gradient alcohol. The heart was embedded with paraffin and then cut into sections of approximately 4 µm thick. The sections were put into xylene and gradient ethanol to dewax, then hematoxylin/eosin staining was performed and they were sealed after dehydration with ethanol. And the slices were observed under an BX-51 light microscope (Olympus, Tokyo, Japan).

### 4.5. RNA-Sequencing Analysis

Four heart tissues in each group were sampled and then frozen in liquid nitrogen and then sent to BGI Co., Ltd., Wuhan, China, for RNA sequencing. Then, 50 mg heart tissue was used to extract the RNA as described in the Extraction of Animal RNA BGI-NGS-TQ-RNA-004 A0. Then, the mRNA were isolated and fragmented, cDNA was synthesized, and a single ‘A’ nucleotide was added to the 3′ ends of the blunt fragments. PCR was conducted and the corresponding library quality control protocol was selected according to the product requirements. Single-stranded PCR products were used to produce single-stranded cyclized products. Single-stranded circle DNA molecules were replicated via rolling cycle amplification, and a DNA nanoball (DNB) which contained multiple copies of DNA was generated. Sufficient-quality DNBs were then loaded into patterned nanoarrays using a high-intensity DNA nanochip technique and sequenced through combinatorial Probe-Anchor Synthesis (cPAS).

The sequencing data was filtered with SOAPnuke and clean reads were obtained and stored in FASTQ format. The subsequent analysis and data mining were performed on Dr. Tom’s Multi-omics Data mining system (https://biosys.bgi.com (accessed on 20 July 2024)). Bowtie2 [28] was applied to align the clean reads to the gene set. The expression level of the genes was calculated by RSEM (v1.3.1) [29]. The heatmap was drawn by pheatmap (v1.0.8) [30] according to the gene expression difference in different samples. Essentially, differential expression analysis was performed using the DESeq2(v1.4.5) [31] with a Q value of ≤0.05 (or FDR ≤ 0.001).

To gain insights into the change in phenotype, GO (http://www.geneontology.org/ (accessed on 30 July 2024)) and KEGG (https://www.kegg.jp/ (accessed on 30 July 2024)) enrichment analysis of annotated different expression gene was performed by Phyper based on a Hypergeometric test. The significant levels of terms and pathways were corrected by Q value with a rigorous threshold (Q value ≤ 0.05) [32].

### 4.6. Protein Extraction and Quantitative Proteomics Based on Data-Independent Acquisition

Four heart tissues were randomly selected in each group and 4% SDS, 100 mM Tris-HCl, pH 7.6, was added to extract the protein. Then the samples were boiled for 15 min and quantified with the BCA Protein Assay Kit (G2026-200T, Servicebio, Wuhan, China). After that, 15 µg of protein from each sample was mixed with 5× loading buffer and boiled for 5 min. The proteins were separated on 4–20% SDS-PAGE gel (constant voltage 180 V, 45 min). Protein bands were visualized by Coomassie Blue R-250 staining. DTT and IAA were added to each sample to block the reduced cysteine residue, and trypsin (trypsin–protein (wt/wt) ratio of 1:50) was added to the sample and incubated at 37 °C for 15–18 h (overnight). The digested peptide was desalted on an MCX desalting column (omicsolution, OS-MCX-1ML), concentrated by vacuum centrifugation, and reconstituted in 20 μL of 0.1% (*v*/*v*) formic acid in water. The peptide content was estimated based on the UV spectral density at 280 nm, and for DIA experiments. The peptides from each sample were analyzed by an OrbitrapTM AstralTM mass spectrometer (Thermo Scientific) connected to a Vanquish Neo system liquid chromatograph (Thermo Scientific) in data-independent acquisition (DIA) mode. Precursor Ions were scanned at a mass range of 380–980 *m*/*z*. The MS1 resolution was 240,000 at 200 *m*/*z*; Normalized AGC Target: 500%; Maximum IT: 5 ms. Next, 299 windows were set to DIA mode in MS2 scanning, with an Isolation Window of 2 *m*/*z*; HCD Collision Energy of 25 ev; a Normalized AGC Target of 500%; and a Maximum IT of 3 ms. Next, during the mass spectrometry data analysis, DIA data was analyzed with DIA-NN 1.8.1. The main software parameters were set as follows: the enzyme used is trypsin; the maximum number of missed cleavages is 1; the fixed modification is carbamidomethyl(C); dynamic modification is performed using oxidation(M) and acetyl (Protein N-term). All reported data were based on 99% confidence for protein identification, as determined by a false discovery rate (FDR) of ≤ 1%.

Cluster 3.0 (accessed on 30 December 2024) and Java Treeview software (http://jtreeview.sourceforge.net (accessed on 30 December 2024)) were used to perform the hierarchical clustering analysis. Protein sequences were searched using the InterProScan 5.6 software to identify protein domain signatures from the InterPro member database Pfam. The protein sequences of the selected differentially expressed proteins were locally searched using the NCBI BLAST+ client software (ncbi-blast-2.2.28+-win32.exe) and InterProScan to find homologue sequences, then gene ontology (GO) terms were mapped and the sequences were annotated using the software program Blast2GO (https://www.blast2go.com/ (accessed on 30 December 2024)). Following annotation steps, the studied proteins were blasted against the online Kyoto Encyclopedia of Genes and Genomes (KEGG) database (https://www.genome.jp/kegg/ (accessed on 30 December 2024)) to retrieve their KEGG orthology identifications and were subsequently mapped to pathways in KEGG. Enrichment analysis was applied based on Fisher’s exact test, considering the whole quantified proteins as the background dataset. And only functional categories and pathways with *p*-values under a threshold of 0.05 were considered significant.

### 4.7. Untargeted Metabolomics Analysis

Six serum samples were randomly selected from each group. The serum samples were stored at −20 °C, and placed in a 4 °C refrigerator to thaw. Then, 100 µL of each sample was added to the EP tube, then the remaining samples were frozen. After that, 700 µL of extractant containing internal standard 1 was shaken for 1 min and placed in −20 °C refrigerator for 2 h. Then, 25,000× *g* was centrifuged at 4 °C for 15 min. The samples were moved out of the centrifuge and 600 µL of the supernatant was transferred to a new split EP tube. Then, 180 µL methanol was added to pure water and vortexed for 10 min, until it had completely dissolved in the reconstituted solution (25,000× *g* centrifuged at 4 °C for 15 min). Next, 20 μL of each sample was mixed with QC samples. The sample extracts were analyzed using Waters UPLC I-Class Plus (Waters, Waltham, MA, USA) equipped with QTRAP 6500 Plus (SCIEX, Boston, MA, USA). After importing the offline data of mass spectrometry into compound discoverer 3.3 (Thermo Fisher Scientific, Waltham, MA, USA) software and analyzing the mass spectrometry data in combination with bmdb (BGI metabolome database), mzcloud database and chemspider online database, a data matrix containing information such as metabolite peak area and identification results was obtained. Bioinformatics analysis mainly included data preprocessing, data quality control [33], overall analysis, screening of differences between groups in comparison groups, analysis of differences between groups in comparison groups, and analysis of differences in multiple comparison groups.

### 4.8. Integration of Transcriptomic, Proteomics, and Metabolic Data

To better understand the common changed pathway in xylazine injection-induced heart injury, we simply combined our xylazine injection groups into a single group. To further elucidate the correlation between the genes and metabolites (or between the proteins and metabolites), the joint pathway analysis of DEGs-DEMs or DEPs-DEMs was performed using the MetaboAnalyst (https://new.metaboanalyst.ca/home.xhtml (accessed on 30 December 2024))). The DEGs and DEMs (or DEPs and DEMs) were simultaneously uploaded into the MetaboAnalyst to analyze the joint pathways enriched by both the DEGs and DEMs (or DEPs and DEMs).

STITCH interaction analysis (accessed on 30 December 2024) was conducted to obtain more detailed information. It is a useful tool for visualizing the associations between functionally linked DEGs, DEPs, and DEMs. The interaction network was processed by Cytoscape (https://cytoscape.org/ (accessed on 30 December 2024)) and the DEGs or DEPs were highlighted with red ellipses while the DEMs were highlighted with orange rectangles. Pathview (accessed on 30 December 2024)) was applied to visualize the matched features in a certain pathway.

### 4.9. Western Blot

Heart tissues were used to prepare the proteins and the sodium dodecyl sulfate (SDS) gels were used to separate the samples at 80 V and 120 V electrophoresis. Then, the target gel bands were transferred to polyvinylidene fluoride (PVDF) membranes at 250 mA for 150 min. The membranes were incubated in 5% skimmed milk for 2 h, and then in diluted primary antibodies against PFKM (1:2000, 55028-1-AP, Proteintech, Rosemont, IL, USA); VDAC3(1:500, 55260-1-AP, Proteintech), GAPDH (1:10,000, 60004-1-Ig, Proteintech), tubulin (1:10,000, GB15140-100, Servicebio) at 4 °C 12 h. They were incubated in Goat Anti-Rabbit IgG H&L/HRP (1:10,000, GB23303, Servicebio) or Goat Anti-Mouse lgG H&L/HRP (1:10,000, GB23301, Servicebio) for 1 h. A chemiluminescence instrument was used to image the bands and Image J v1.8.0.345 was used to calculate the gray values.

### 4.10. Statistical Analysis

All results are presented as mean ± SEM. The statistical analyses were performed using SPSS 26.0 through one-way ANOVA and Tukey’s test, and a *p* of less than 0.05 between different group was considered indicative of a significant difference. The pictures were drawn using Graphpad Prism 10.1.

## 5. Conclusions

Xylazine-induced heart injury was confirmed through changes in cardiac ultrasound, cardiac enzymes, and histopathological examination. The altered gene, protein, and metabolite levels were meticulously described using transcriptomic, proteomic, and metabolic analyses. The integration of multi-omics networks revealed 25 overlapping pathways between differentially expressed genes and metabolites (DEGs-DEMs) and differentially expressed proteins and metabolites (DEPs-DEMs), with most related to sugar, amino acid, and fat metabolism. This confirmed the crucial role of energy metabolism in xylazine-induced heart injury. The proteins involved in fructose and mannose metabolism, as well as cholesterol metabolism pathways, were verified, thus confirming their critical role in the process of xylazine-induced heart injury. Further studies are still needed to elucidate the underlying mechanisms of xylazine-induced heart injury.

### Limitations

Although we described the transcriptomic, proteomic, and metabolic changes in xylazine-induced heart injury, the limitations of the present study should also be emphasized. Firstly, the animal model of repeated xylazine injections was established based on our previous study; whether it can mimic xylazine poisoning in the real world still requires further investigation. Secondly, the transcriptomic, proteomic, and metabolic changes in xylazine-induced heart injury were carefully described in the present study, and the integration of the transcriptomic, proteomic, and metabolic networks was also conducted. However, the results of the present study still need vitro or in vivo experiments to clarify the underlying mechanism, which limits the significance of the pathways found. Finally, in clinical and forensic practice, xylazine poisoning is often combined with other drugs. The present study focused solely on the role of xylazine in the animal model; the interactions between xylazine and other drugs still require further study to clarify.

## Data Availability

The original contributions presented in this study are included in the article/Supplementary Materials. Further inquiries can be directed to the corresponding author.

## Supplementary Materials

The following supporting information can be downloaded at https://www.mdpi.com/article/10.3390/ijms26178532/s1.
