# Supplementary figures and images for: Integrated Multilayer Omics Reveals the Underlying Mechanisms in Xylazine-Related Heart Injury in Rats
Source: Int J Mol Sci. 2025 Sep 2;26(17):8532. doi: 10.3390/ijms26178532 (PMC12428804; doi:10.3390/ijms26178532)

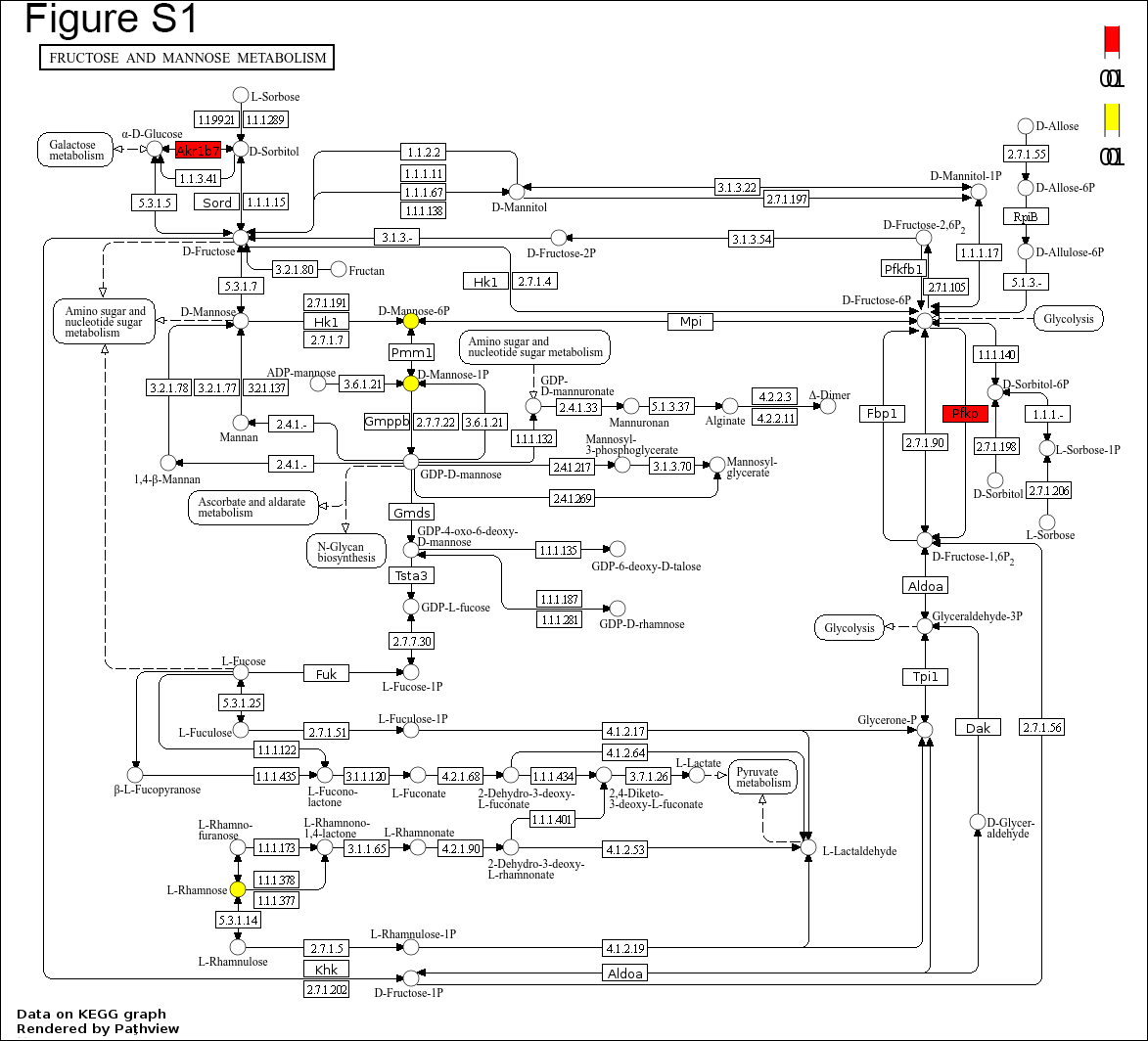

Supplement: Supplementary file 1 [file ijms-26-08532-s001.zip › Figure S1.tif]

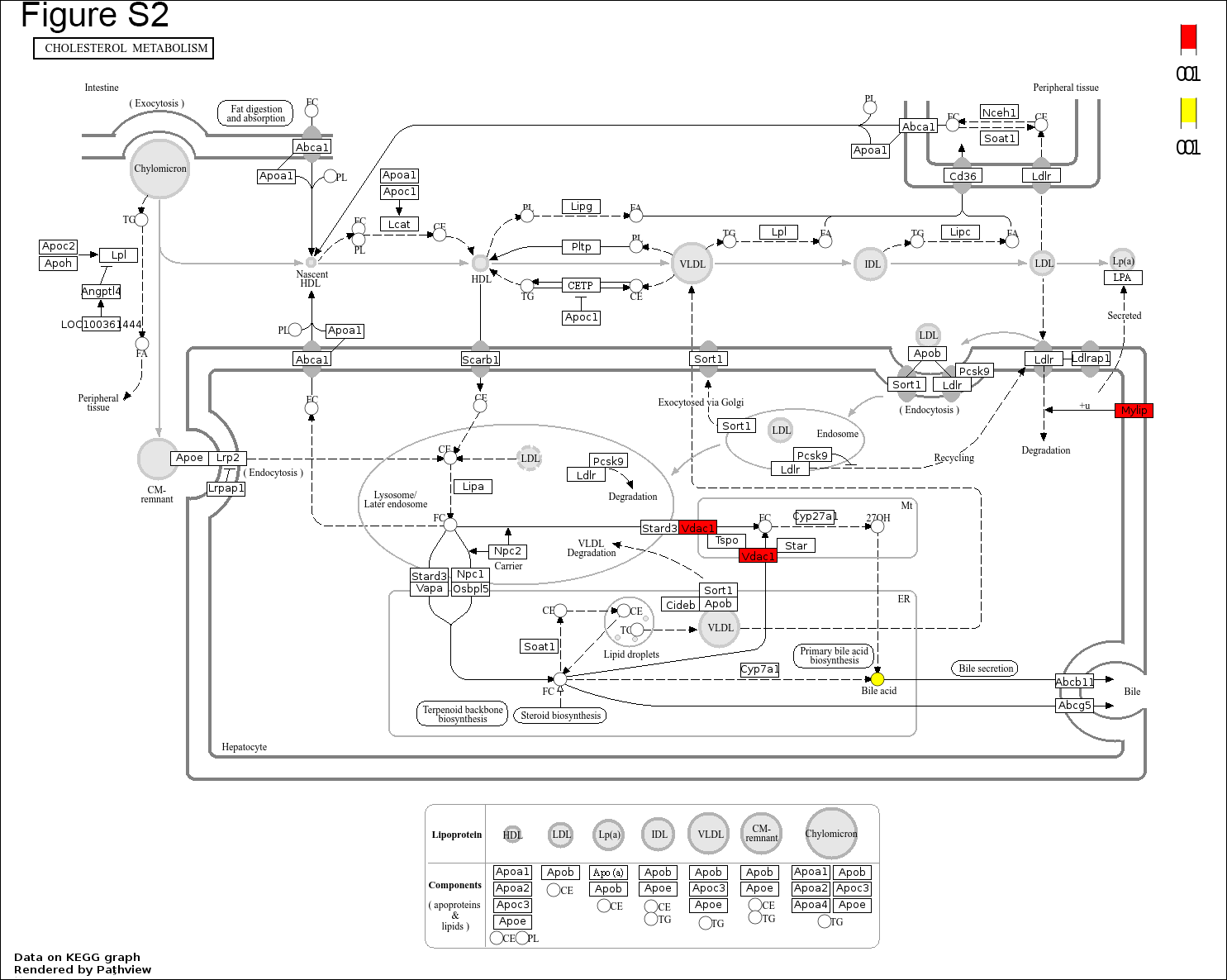

Supplement: Supplementary file 1 [file ijms-26-08532-s001.zip › Figure S2.tif]
